# Supplementary material for: Functional Analysis of V2 Protein of Beet Curly Top Iran Virus
Source: Plants (Basel). 2022 Dec 2;11(23):3351. doi: 10.3390/plants11233351 (PMC9736138; doi:10.3390/plants11233351)
Supplement: Supplementary file 1 [file plants-11-03351-s001.zip › plants-1955718-supplementary.pdf]

```
                                                                NLS
                   10        20        30        40        50         60
BCTIV  MVPFRVSEFPRSYPALLAVSTSCFLRYNKWCLLGIEPQVREPLTLEEGEDFLQFQKEVKKL
BCTV   MGPFRVDQFPDNYPAFLAVSTSCFLRYNRWCILGIHQEIGSLTLEEGEVF-RQFQKEVKKL

                 NLS
                70        80        90       100       110       120
BCTIV  LKLKVSFKRKCSLYEDIYKKYGGSESKEKVYSSGQLDEEEEDDRWEDSFEEIPMEETGAK
BCTV   LRCKVNFHRKCSLYEEIYKKYVYNVPEKKGESSKCVAEEEED
```

Supplementary Figure S1: Alignment of the aminoacid sequences of the V2 proteins from the becurtovirus beet curly top Iran virus (BCTIV; AFK14083) and the curtovirus beet curly top virus (BCTV; M24597). Predicted Nuclear localization signal (NLS) are depicted in white letters inside black boxes.

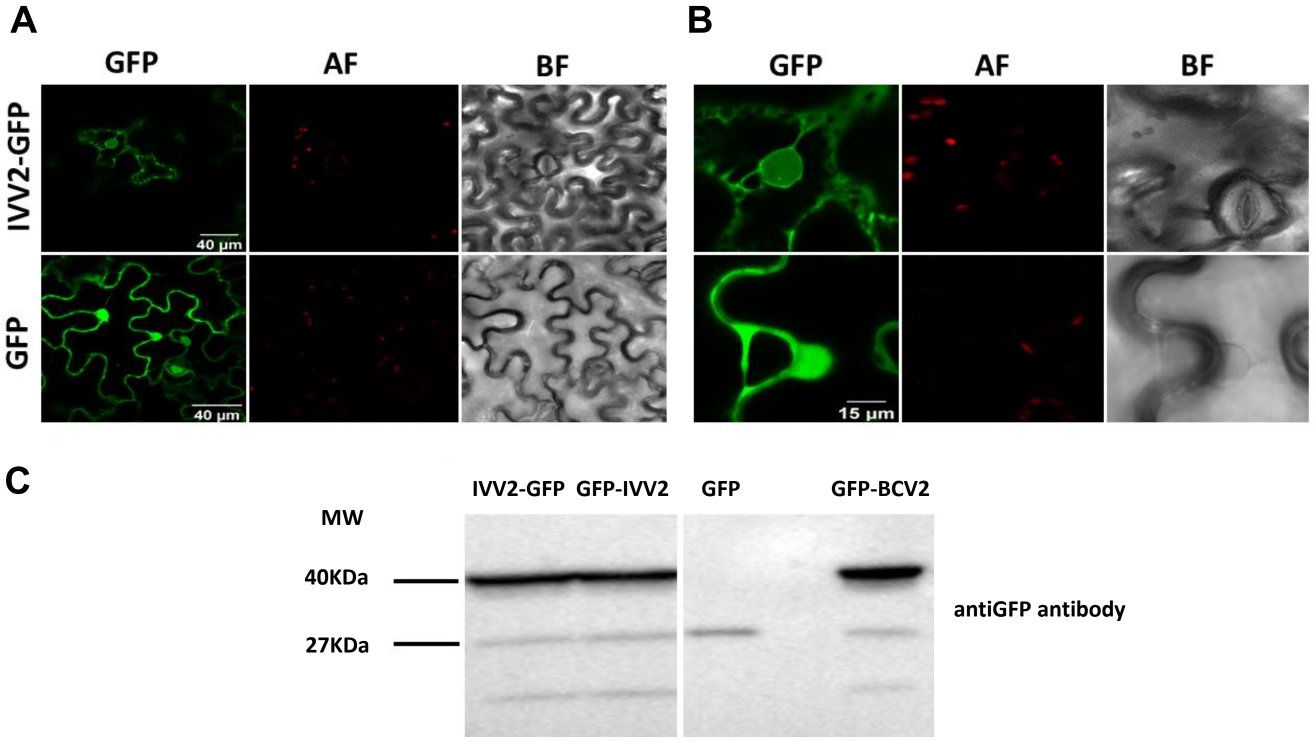

Supplementary Figure S2: Subcellular localization of V2 from beet curly top Iran virus fused to GFP in epidermal cells of *Nicotiana benthamiana*. (A) Leaves were agroinfiltrated with a construct expressing the 35S:GFP (GFP), 35S:GFP-V2 or 35S:V2-GFP fusion proteins from beet curly top Iran virus (GFP-IVV2 or IVV2GFP respectively) or 35S:GFP-V2 fusion protein from beet curly top virus (GFP-BCV2). Samples were observed under the confocal microscope at 36 hours post infection. (B) Close up confocal images of the observed areas. GFP fluorescence (GFP), autofluorescence (AF) and the bright field channel (BF) are shown (C) western blot analysis of the observed tissues with antiGFP antibody.

```
BCTIV     MMVCIPDWLFLLFVFSTILQSGINFYGTFQSERISAKLSSLASRFDELFLALQQVVFARN   60
LaaV      MVVCMPDWLFLVFIFGSIAQSGISLYGTIQSKGFSRHLSEISSSLHSLFASVEQVLHTWD   60
EmaV      MMVCLPDWLFLLLILSILVQAAVGFYGSFQGERFSSKLSGISSRCDELFVALQQVVYSRN   60
OpV2      MMVCIPDWLFLLFIFSVLLDSTVIFYGTFQSGSISQKLSILSSRVDELFLAIQQVVHSRC   60
HrCTV     MMVCLPDWVFLLFIASILLQACTNFYGTFHSGSISKKLSSLVSRFDELFFQIQQVVYTRY   60
SCTAV     MMVCLPDWLFLLFILSILLQSGTNFYGTFQSGTVSKQLSCVASRVDELFLQIQQVVYSRN   60
SSCTV     MMVCLPDWLFLLFIFAILLQSGTNFYGTIQSGSISRKLSSLSSRFDELFLKIQQVVYIRS   60
PeYDV     MMVCLPDWLFLLFIFSILLQSGTNFYGTFQSGSISRQLSSLSSRIDQLFLKIQQVVYTRY   60
BMCTV     MMVCLPDWLFLLFIFSILLQSGTNFYGTFQSGSISRQLSSLSSSIDQLFLKVQQVVYTRY   60
BCTV      MMVCLPDWLFLLFIFSILLQSGTNFYGTFQSGSISRQLSSLSSSIDQLFLKVQQVVYTRY   60
BSCTV     MMVCLPDWLFLLFIFSILLQSGTNFYGTFQSGSISRQLSSLSSSIDQLFLKVQQVVYTRY   60
          *:**:***:**::: . : ::   :**:::.  .* :** : *  ..**  ::**:.   |

BCTIV     RVSSSSRAVDSRGRRGLSSIPEGSEEIAEA--   89
LaaV      T-VGIRRVRPRRRGGLSSVQEGGPETPTEEG   90
EmaV      ASRGNRNPYCRRAGELYTVPEAGEENPQV--   89
OpV2      A-TRDRTADFRRRRSLSAIPEGSEEIAEAQV   90
HrCTV     P-SRSRIVNPRRRGSLSGIPEGGEETVEA--   88
SCTAV     T-AGNRAVESRRRRGLSSVPEGSEEVAEA--   88
SSCTV     N-TRDRGVDTRRRRGLSSIPEGSEEVAEA--   88
PeYDV     P-SRDRATDPRRRRGLSSIPEGSEEVAEA--   88
BMCTV     P-SRDRASDPRRRRGLSAIPEGSEEATEV--   88
BCTV      P-SRDRVSDPRRRRGLSSIPEGSEEATEV--   88
BSCTV     P-SRDRGSDPRRRRGLSSIPEGSEEATEA--   88
               *     *    *  : *..  *
```

Supplementary Figure S3: Alignment of the aminoacid sequences of the V3 proteins from the geminiviruses: beet curly top Iran virus (BCTIV; YP_001715617.1), Limeum africanum associated virus (LaaV; YP_009465967.1), Exomis microphylla associated virus (EmaV; YP_009465978.1), Opuntia virus 2 (OpV2; QTT61872.1), horseradish curly top virus (HrCTV; NP_066181.1), spinach curly top Arizona virus (SCTAV; YP_004207921.1), Spinach severe curly top virus (SSCTV; YP_003966133.1), pepper yellow dwarf virus-Mexico (PeYDV; YP_009362973.1), beet mild curly top virus (BMCTV; AAN06926.1), beet curly top virus (BCTV; NP_899663.1) and beet severe curly top virus (BSCTV; AAA20511.1) Changed aminoacid is highlighted in grey and bold letters.

Table S1: Primer used in this study

Name	Sequence from 5' to 3'	Reference	Use
BIV2Fw	AAAGCAGGCTTCATGGTACCTTTCAGAGTGAGC	This study	Generation of entry clone pDONR-zeo-IVV2
BIV2RvSt	GAAAGCTGGGTCTTACTTCGCACCGGTCTC		
BIV2RvNoSt	GAAAGCTGGGTCCTTCGCACCGGTCTC		
*Cla*I V2 F	CCATCGATATGGTACCTTTCAGAGTGAGC	This study	Generation of PVX-IVV2 and Semiquantitative RT-PCR to detect BCTIV V2 mRNA
*Sal*I V2 R	ACGCGTCGACTTACTTCGCACCGGTCTC		
IVV2 stpF	CTTTCAGAGTGAGCTAATTTCCGCGAAGC	This study	Site-directed mutagenesis of V2 ORF in BCTIV 1.3mer infectious clone
IVV2 stpR	GCTTCGCGGAAATTAGCTCACTCTGAAAG		
BCTIV-RTF	TGGCTAGTGGTGCATTTTGG	This study	q-PCR to quantification BCTIV DNA
BCTIV-RTR	CGCATCCCTCCTAATCCGAT		
LowEF-1a	AGCTTCGTGGTGCATCTC	Rotenberg et al., 2006	Semiquantitative RT-PCR and q-PCR normalizer gene
UpEF-1a NB	GATTGGTGGTATTGGAACTGTC		
LowBCTV-qRT	CTACACGAAGATGGGCAACCT	Luna et al., 2017	Semiquantitative RT-PCR to detect BCTV V2 mRNA
UpBCTV-qRT	TGACGTCGGAGCTGGATTTAG		
